# Risk-Sensitive Decision-Making in Patients with Posterior Parietal and Ventromedial Prefrontal Cortex Injury

**DOI:** 10.1093/cercor/bht197

**Published:** 2013-08-07

**Authors:** Bettina Studer, Facundo Manes, Glyn Humphreys, Trevor W. Robbins, Luke Clark

**Affiliations:** 1 Department of Psychology; 2 Behavioural and Clinical Neuroscience Institute (BCNI), University of Cambridge, Cambridge, UK,; 3 Institute of Cognitive Neuroscience, University College London, London WC1N 3AR, UK; 4 Institute of Cognitive Neurology (INECO), Favaloro University, Buenos Aires, Argentina and; 5 Department of Experimental Psychology, University of Oxford, Oxford, UK

**Keywords:** decision-making, lesion study, posterior parietal cortex, risk, ventromedial prefrontal cortex

## Abstract

Successful choice under risk requires the integration of information about outcome probabilities and values and implicates a brain network including the ventromedial prefrontal cortex (vmPFC) and posterior parietal cortex (pPAR). Damage to the vmPFC is linked to poor decision-making and increased risk-taking. Electrophysiological and neuroimaging data implicate the pPAR in the processing of reward probability during choice, but the causal contribution of this area has not been established. We compared patients with lesions to the pPAR (*n* = 13), vmPFC (*n* = 13), and healthy volunteers (*n* = 22) on the Roulette Betting Task, a measure of risk-sensitive decision-making. Both lesion groups were impaired in adjusting their bets to the probability of winning. This impairment was correlated with the extent of pPAR, but not vmPFC, damage. In addition, the vmPFC group chose higher bets than healthy controls overall, an effect that correlated with lesion volume in the medial orbitofrontal cortex. Both lesion groups earned fewer points than healthy controls. The groups did not differ on 2 tasks assessing probabilistic reasoning outside of a risk-reward context. Our results demonstrate the causal involvement of both the pPAR and vmPFC in risk-sensitive choice and indicate distinguishable roles of these areas in probability processing and risk appetite.

## Introduction

Day-to-day decision-making involves frequent choices between options with uncertain outcomes, and successful decision-making under uncertainty is thought to involve an interplay combination of cognitive and emotional processes (e.g. Damasio 1996; Dolan 2002; Coricelli et al. 2007; Kahneman 2011). Decision-making is implemented by a widespread network of brain areas, comprising frontal, parietal, and subcortical structures (e.g. Ernst and Paulus 2005; Krain et al. 2006; Platt and Huettel 2008; Vorhold 2008; Kable and Glimcher 2009; Liu et al. 2011). Within this network, the emotional processing of decision information has been proposed to rely particularly on the ventromedial prefrontal cortex (vmPFC; e.g. Damasio 1994, 1996). Patients with vmPFC damage display increased risk-taking on laboratory gambling tasks: These cases fail to learn the advantageous strategy on the Iowa Gambling Task, make more risky bets on the Cambridge Gamble Task, and prefer immediate rewards over larger delayed rewards on a delay discounting task (Bechara et al. 1994, 1999, 2000; Mavaddat et al. 2000; Manes et al. 2002; Bechara et al. 2005; Fellows and Farah 2005; Weller et al. 2007; Clark et al. 2008; Sellitto et al. 2010). However, the precise mechanism underlying this change in risky choice is less clear. Are these patients unable to adequately process or integrate gain and loss information, or might they genuinely prefer the riskier options (Sanfey et al. 2003; Clark et al. 2008)?

The posterior parietal cortex (pPAR) is also implicated in decision-making (for reviews, see Krain et al. 2006; Platt and Huettel 2008; Kable and Glimcher 2009). Electrophysiological studies in nonhuman primates found that firing rates of pPAR neurons correlate with the probability of reward during response selection in binary choice situations, including perceptual decision-making (e.g. Platt and Glimcher 1999; Shadlen and Newsome 2001; Yang and Shadlen 2007; Kiani and Shadlen 2009). Recent functional magnetic resonance imaging (fMRI) work in humans demonstrates that activity in the pPAR during decision-making is sensitive to the probability and variance of uncertain outcomes (Huettel et al. 2005; Van Leijenhorst et al. 2006; Smith et al. 2009; Vickery and Jiang 2009; Symmonds et al. 2011; Studer et al. 2012). Taken together, this research suggests a crucial role of the pPAR in decision-making, linked to the processing of outcome probabilities, but this conclusion is yet to be substantiated with causal methodologies.

**Figure 1.**
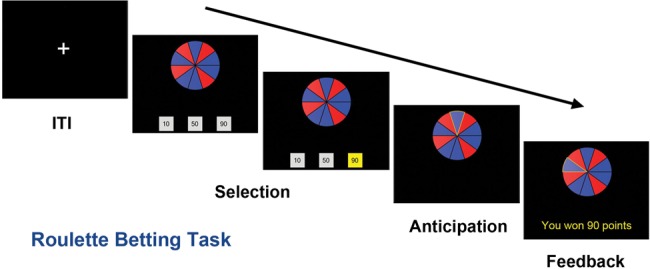
Roulette Betting Task. Participants were presented with a wheel containing 10 blue and red segments. Blue segments were defined as winning, and red segments as losing, and the proportion of blue segments varied across trials (40%, 60%, or 80%). On active-choice trials (shown), the participant selected 1 of the 3 available bets (10, 50, or 90 points), while in the no-choice control condition, the 3 bet options were of the same amount.

In the present study, patients with damage to the pPAR (*n* = 13) and vmPFC (*n* = 13), as well as healthy control participants (*n* = 22), were administered the Roulette Betting Task (RBT; Studer and Clark 2011; Studer et al. 2012) to quantify their risk-sensitive decision-making. This task was derived from the Cambridge Gamble Task, and retains the key feature that participants select bets on risky gambles across varying chances of winning (Fig. 1). Unlike the Cambridge Gamble Task, the RBT assesses decision-making under both positive odds, when risk-taking is advantageous, and negative odds, when conservative betting is optimal. We predicted that impaired decision-making would be evident in both lesion groups. Furthermore, the RBT allows the separation of the overall level of betting (“risk appetite”), from the degree to which bets are adjusted to the probability of winning (“risk adjustment”). [The use of this term highlights a subtle discrepancy between economic definitions of risk as peak uncertainty (e.g. Preuschoff et al. 2006) and psychological definitions of risk as potential for loss (e.g. Slovic 1987; Weller et al. 2007). We adopted the term risk adjustment from the previous literature on the Cambridge Gamble Task (e.g. Rogers et al. 1999; Deakin et al. 2004; Clark et al. 2008; Newcombe et al. 2011), and use it to refer to the level of betting as a function of the chances of winning/losing.] We hypothesized that vmPFC damage would primarily affect risk appetite, whereas patients with pPAR damage would primarily manifest reduced risk adjustment.

Group comparisons were supplemented with region-of-interest (ROI) analyses performed on structural MR data, which correlate these 2 parameters (risk adjustment and overall betting) against the extent of damage in the pPAR and the vmPFC. Within each sector, 2 functionally significant subdivisions were identified: The inferior and superior parietal cortices (IPC and SPC; Cavada and Goldman-Rakic 1989), and the medial orbitofrontal and medial prefrontal cortices (mOFC and mPFC; Rushworth et al. 2006, 2007; Wallis 2007; Rushworth and Behrens 2008). Furthermore, 2 control tasks measuring probabilistic reasoning (the Beads Game; Huq et al. 1988; Garety et al. 2005) and the processing of probabilities outside of a gambling context (the Probability Adjustment Task; Falk and Wilkening 1998) were administered. The inclusion of these tasks enabled us to test whether basis understanding of odds was intact, and whether the expected impairment in using probability information for response was specific to a risk-reward context.

## Materials and Methods

### Participants

Three groups of participants took part in this study: Neurological patients with focal lesions to (1) the pPAR (*n* = 13), (2) the vmPFC (*n* = 13), and (3) healthy controls subjects (*n* = 22). All lesion patients were recruited from the Cambridge Cognitive Neuroscience Research Panel at the MRC Cognition and Brain Sciences Unit, Cambridge, with the exception of 3 pPAR lesion patients recruited from a panel at the Behavioural Brain Sciences Centre, University of Birmingham. All patients had stable, adult-onset lesions and were in the chronic phase of recovery (lesion sustained at least 3 years prior to testing).

**Figure 2.**
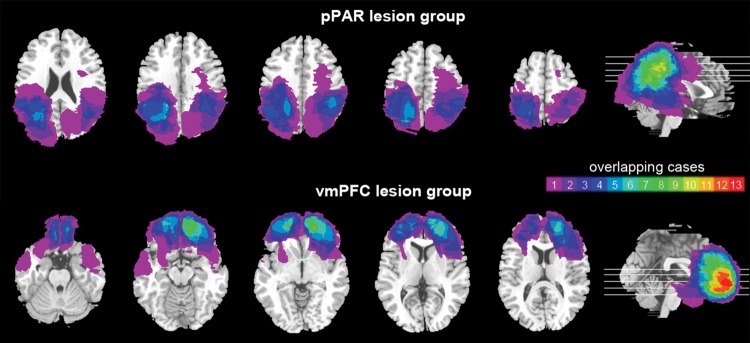
Lesion overlap in the pPAR lesion group (top panel) and vmPFC lesion group (bottom panel). The color bar indicates the number of overlapping cases at each voxel.

Lesion location was confirmed from the lesion overlap with anatomical regions defined from the Automatic Anatomical Labeling (AAL) atlas (Tzourio-Mazoyer et al. 2002). The pPAR group had sustained damage to at least one of: Angular gyrus, supramarginal gyrus, inferior parietal lobe, superior parietal lobe, or precuneus (see Fig. 2, top panel). Some patients also had damage to areas adjacent to the pPAR, but critically, their lesions spared the prefrontal cortex. The pPAR group included a mixture of bilateral (*n* = 3), left unilateral (*n* = 7), and right unilateral (*n* = 3) lesions, and lesion etiology was tumor resection (*n* = 5), ischemic or hemorrhagic stroke (*n* = 5), infarct (*n* = 1), or hemorrhage (*n* = 2). The vmPFC group had sustained damage to at least one of: The gyrus rectus, the orbital parts of the middle and superior frontal gyri, medial superior frontal gyrus, or anterior cingulate cortex (see Fig. 2, lower panel). Some lesions extended into areas adjacent to the vmPFC, but importantly all lesions in the vmPFC group spared the parietal lobe. The vmPFC group consisted of bilateral (*n* = 6), right unilateral (*n* = 4), and left unilateral (*n* = 3) lesions, and lesion etiology was hemorrhage (*n* = 6) or tumor resection (*n* = 7).

Healthy participants were recruited from the local community. Exclusion criteria included a history of psychiatric or neurological disease, regular use of psychoactive drugs, and regular gambling. The healthy control group did not differ significantly from the patient groups in age (*F* = 0.88, *P* = 0.42), gender (*X*^2^ = 2.54, *P* = 0.28), years of education (*F* = 0.42, *P* = 0.66), or estimated premorbid intellectual functioning (*F* = 0.57, *P* = 0.57; see Table 1), assessed with the National Adult Reading Test (NART; Nelson and Willison 1991). The chronicity of vmPFC and pPAR lesion patients did not significantly differ (*T* = 0.42; *P* = 0.68). Self-reported trait impulsivity, assessed with the Barratt Impulsiveness Scale (Patton et al. 1995), was somewhat higher in pPAR lesion patients than in healthy controls (*P* = 0.02) and vmPFC lesion patients (*P* = 0.1), while vmPFC lesions patients and healthy controls did not significantly differ (*P* = 0.47; see Table 1).

**Table 1:** Background information on the 3 groups of participants

Group	Gender (f/m)	Age	Years of education	NART score	Barratt score	Chronicity (months)
vmPFC	8/5	58.2 (±12.3)	13.5 (±2.6)	116.3 (±7.2)	61 (±11)	122.6 (±46.2)
pPAR	4/9	63.3 (±8.7)	13.4 (±3.6)	116.1 (±10.5)	67 (±10)	114.1 (±58)
Controls	11/11	60.1 (±8.7)	14.2 (±2.5)	118.7 (±6.7)	58 (±8)	n.a.

Cells show means and standard deviations.

n.a.: not applicable.

Participants took part in a single testing session lasting approximately 2h. All participants completed the RBT. The 2 control tasks could not be administered to all participants, due to time constraints. Tasks were programmed in Visual Basic (Microsoft Corporation, USA) and run on a laptop computer. This study was approved by the National Research Ethics Committee. All participants provided written informed consent, in accordance with the Declaration of Helsinki. Participants received £15 for participation plus a variable bonus depending on their score in the RBT, which could range between £0 and £5.

### Neuroanatomical Analysis

Lesion locations were confirmed using MRI with a 1.5-T scanner. Lesions were traced on the structural scan for each patient by an experienced neurologist (F.M.) who was blind to task performance, using the MRIcro software (Chris and Matthew 2000). Structural scans were then normalized to the MNI305 template using SPM99 (Statistical Parametric Mapping; Wellcome Department of Cognitive Neurology, London, UK) with cost function masking applied to exclude the lesion in the calculation of normalization parameters (Brett et al. 2001). MRIcro software was used to create the lesion overlay for the 2 patient groups and calculates the volume of total brain damage. The 2 patient groups did not differ in the volume of total lesion (*P* = 0.78; vmPFC group: *M* = 47 504 voxels, pPAR group: *M* = 50 192 voxels).

### Roulette Betting Task

The Roulette Betting Task was used to assess risk-sensitive decision-making (Studer and Clark 2011; Studer et al. 2012). In each trial, participants were presented with a wheel containing 10 red and blue segments, and 3 bet options (see Fig. 1). The ratio of blue (winning) to red (losing) segments varied across trials, reflecting the chances of winning (40%, 60%, or 80%). The wheel was presented centrally, with winning segments distributed evenly across the left and right sides of the wheel. On each trial, participants selected 1 of the 3 available bet options with a key press. On active-choice trials, the available bets were 10, 50, and 90 points. On no-choice trials, the 3 bet boxes contained identical amounts, serving as a control condition to measure psychomotor slowing. Bet selection was self-paced. Next, the wheel spun for a variable anticipation period (3–3.5 s) and then stopped on 1 of the 10 segments. If the wheel stopped on a blue segment, the chosen bet was won and win feedback was presented. If the wheel stopped on red, the bet was lost and loss feedback appeared. A variable intertrial interval (2–2.5 s) presented a fixation cross. Participants completed 3 practice trials, followed by a total of 90 trials (45 active-choice and 45 no-choice), divided into 3 blocks of 30 trials each. At the end of each block, the current point score was presented, and participants took a short break to avoid fatigue.

Betting behavior was extracted from the active-choice trials, with 2 parameters calculated reflecting (1) overall betting (average selected bet amount), (2) risk adjustment, formalized as the slope of the best line of fit through the average bet on 40%, 60%, and 80% trials. Response times were analyzed including active-choice and no-choice trials in repeated-measures analysis of variance (ANOVA), and the final point score was also extracted as an overall performance metric.

### Control Tasks

In addition to the RBT, we administered 2 control tasks assessing probability processing outside of a risk-reward context. Both control tasks involved handling probabilistic information, but did not involve betting, wins, or losses. The first control task, the Probability Adjustment Task, was a computerized version of a test developed in child psychology by Falk and Wilkening (1998). It measures the ability of individuals to understand and calculate odds across different levels of difficulty. On each trial, 2 urns were presented; the “full urn” and the “target urn” (see Supplementary Fig. 1 for a screen shot). The full urn contained red and blue beads, and the target urn contained only red beads. Participants were instructed to add blue beads to the target urn in order to create equal chances of drawing a red bead from either urn; that is, to match the proportion of blue-to-red beads in the 2 urns. The total number of beads, and the ratio of red-to-blue beads, varied across trials, such that 3 levels of difficulty varying in the complexity of the required mathematical calculations were presented. Two practice trials were followed by 8 experimental trials. No feedback was presented. Correct responses were analyzed across the 3 levels of difficulty and overall. The task was administered to 11 vmPFC lesion patients, 11 pPAR lesion patients, and 22 healthy controls. The task was aborted in 3 pPAR lesion patients who unable to adequately count the beads. These subjects were excluded from analysis, and the final sample consisted of 11 vmPFC lesion patients, 8 pPAR lesion patients, and 22 healthy controls.

The second control task, the Beads Game, is a test of probabilistic reasoning and information sampling, widely used in the investigation of delusion formation (Huq et al. 1988; Garety et al. 2005). We administered the task to assess individual's to make judgments based on probabilistic information when no risky outcomes were involved. On each trial, participants were shown 2 jars, each containing 100 green and red beads. The ratio of green-to-red beads (60:40 or 85:15) was inverted in the 2 jars. Participants were informed that the computer had randomly picked 1 of the 2 jars and would now draw beads from that jar one at a time. Each drawn bead was then replaced, so that the overall proportion of red-to-green beads in the jars did not change across trials. Previously drawn beads were displayed throughout the trial, to reduce working memory demands, and the sequence of drawn beads was fixed across all participants. Participants completed 4 trials each of 2 conditions (see Supplementary Fig. 2 for screen shots). In the “draws to decision” condition (administered first), participants were asked to decide which jar the beads were being taken from, with the instruction that they could draw as many beads as they required to make their decision. The dependent variable was the number of requested beads. In the “probability estimation” condition, participants were presented with 10 consecutive draws, and after each draw they rated the likelihood that the beads were coming from a given jar. The ratings after the 1st draw (“initial likelihood”) and 10th draw (“final likelihood”) were assessed, as well as the change in rating following beads of the color opposite to the current majority (“response to disconfirmatory evidence”). The task was completed by 9 pPAR lesion patients, 12 vmPFC lesion patients, and 20 healthy controls.

### Data Analysis

The RBT and the Beads Game were analyzed with mixed-model ANOVA, with group as a between-subjects factor and task measures (e.g. probability of winning) as within-subject factors. Greenhouse-Geisser correction was applied when homogeneity of variance was violated. Significant main effects of group were followed up by pair-wise comparisons using Fisher's least significant difference, which is suitable for post hoc testing in cases with 3 experimental groups (Cardinal and Aitken 2006). Effect sizes for pair-wise comparisons were computed using Cohen's *d*. As the data on the Probability Adjustment Task were noncontinuous, these were analyzed with nonparametric tests (Kruskal–Wallis tests). All statistical tests are reported 2-tailed and *α* was set at 0.05.

In a second step, significant effects of group were followed up by ROI analysis. The extent of damage within predefined ROIs was computed. ROIs were defined using the AAL template. For each vmPFC lesion patient, the volume of damage in the vmPFC as a whole, in the mOFC (AAL regions: Gyrus rectus and orbital parts of the middle and superior frontal gyri), and mPFC (AAL regions: anterior cingulum and medial superior frontal gyrus) subregions was calculated. For each pPAR lesion patient, the volume of damage in the pPAR as a whole, in the IPC (AAL regions: Inferior parietal lobe, angular gyrus, and supramarginal gyrus), and SPC (AAL regions: Superior parietal lobe and precuneus) subregions was calculated. Spearman's correlations were then calculated between these lesion volumes and the 3 behavioral indices on the RBT (final score, risk adjustment, and overall betting).

## Results

### Betting Behavior

**Figure 3.**
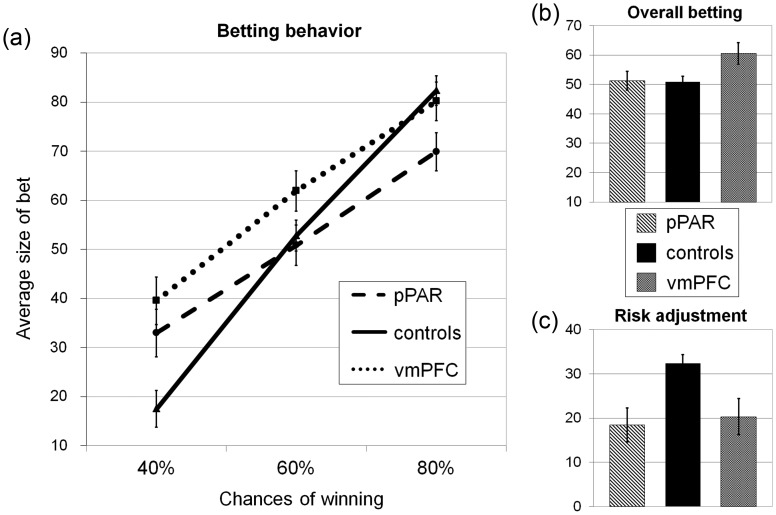
Betting behavior. (*a*) Average bet size in 40%, 60%, and 80% trials for each of the 3 experimental groups. Compared with healthy controls, pPAR lesion patients adjusted their bets less to the chances of winning, choosing larger bets at low chances of winning and smaller bets at high chances of winning. vmPFC lesion patients selected higher bets than healthy controls overall, and particularly at low chances of winning. (*b*) Overall betting behavior collapsed across the 3 chances of winning in the 3 groups: vmPFC lesion patients selected higher bets overall than healthy controls (*P* = 0.02) and pPAR lesion patients (*P* < 0.05). (*c*) Risk adjustment scores in the 3 groups: Decreased risk adjustment scores were observed in both patient groups compared with healthy control subjects (*P* < 0.01). Error bars represent SEM.

A mixed-model ANOVA was run on the average bet size, with chances of winning as a within-subjects factor. There was a significant main effect of the chances of winning (*F*_2,90_ = 137.84, *P* < 0.001, ηp^2^ = 0.75), such that bet size increased with the likelihood of winning, and a significant main effect of group (*F*_2,45_ = 3.64, *P* < 0.05, ηp^2^ = 0.14), with the vmPFC group choosing higher bets than the pPAR group (*P* < 0.05, *d* = 0.79) and the healthy control group (*P* = 0.01, *d* = 0.93), who did not differ (*P* = 0.93, *d* = 0.03, see Fig. 3*b*). The interaction of group by chances of winning was also significant (*F*_4,90_ = 5.64, *P* < 0.001, ηp^2^ = 0.20, see Fig. 3*a*), and we conducted 2 sets of follow-up comparisons to explore this interaction term. First, we compared the degree to which each group adjusted their bets to the probability of winning, running a 1-way ANOVA on the risk adjustment measure: There was a significant effect of group (*F*_2,47_ = 7.28, *P* < 0.01), with reduced risk adjustment in both lesion groups compared with healthy controls (pPAR vs. controls: *P* < 0.01, *d* = 1.23; vmPFC vs. controls *P* < 0.01, *d* = 1.01; see Fig. 3*c*), and no difference between the pPAR and vmPFC groups (*P* = 0.69, *d* = 0.13).

Secondly, we compared betting behavior for the 40%, 60%, and 80% trials separately, using 1-way ANOVAs. On 40% trials, the main effect of group was significant (*F*_2,47_ = 7.34, *P* = 0.002), with both lesion groups selecting higher bets than the healthy control group (vmPFC vs. controls: *P* = 0.001, *d* = 1.33; pPAR vs. controls: *P* = 0.01, *d* = 1.33), and no difference between the lesion groups (*P* = 0.34, *d* = 0.32). On 60% trials, the effect of group was nonsignificant (*F*_2,47_ = 2.16, *P* = 0.13). In 80% trials, a significant main effect of group was identified (*F*_2,47_ = 3.32, *P* < 0.05), with pPAR lesion patients selecting lower bets than both the healthy control group (*P* = 0.015, *d* = 0.90) and vmPFC lesion group (*P* = 0.07, *d* = 0.66), who did not differ (*P* = 0.66, *d* = 0.18). Notably, most participants in the vmPFC and healthy control groups selected the highest possible bet (90 points) in all 80% trials, creating a ceiling effect that may have obscured a further elevation in betting and driven the apparent reduction in risk adjustment in the vmPFC.

In summary, pPAR lesion patients showed impairment in adjusting their bets to the chances of winning, selecting higher bets than healthy controls when the chances of winning were unfavorable but lower bets when the chances of winning were strongly favorable. Patients with vmPFC damage showed increased betting compared with healthy controls when the chances of winning were unfavorable.

### Final Point Score

There was a significant difference in final points won on the RBT (*F*_2,47_ = 7.42, *P* = 0.002; see Table 2), with both pPAR lesion patients and vmPFC lesion patients achieving lower scores than healthy controls (vmPFC vs. controls: *P* = 0.01, Cohen's *d* = 1.04; pPAR vs. controls: *P* = 0.001, *d* = 1.28), while the 2 lesion groups did not significantly differ (*P* = 0.42, *d* = 0.30).

**Table 2:** Overall decision-making performance of the 3 groups of participants

Group	Final score		Comparisons	
	Mean	SEM	Direction	*P*-value
pPAR	1058	66	pPAR < controls	0.001
Controls	1311	38	vmPFC < controls	0.01
vmPFC	1123	55	pPAR vs. vmPFC	0.42

### ROI Correlations

There were no significant correlations observed between the 3 behavioral indices (overall betting, risk adjustment, and final point score) and the total lesion volumes (all *P* > 0.2).

**Figure 4.**
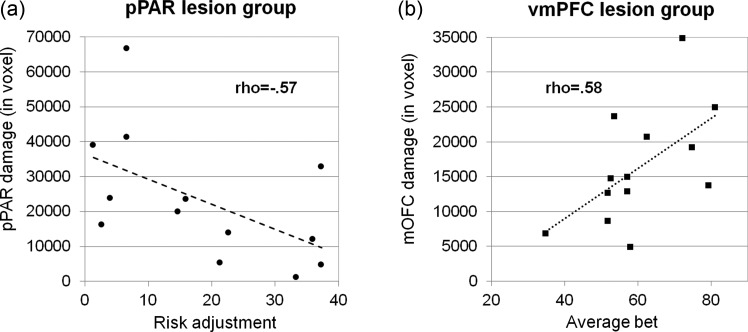
Relationships between betting behavior and lesion volume in the pPAR and mOFC. (*a*) In the pPAR lesion group, overall pPAR lesion volume predicted lower risk adjustment. (*b*) In the vmPFC lesion group, the volume of damage in the mOFC subregion predicted higher overall betting.

In the pPAR group, a significant negative correlation was found between risk adjustment and the volume of pPAR damage (*ρ* = −0.57, *P* = 0.04, *n* = 13): Patients with greater pPAR damage adjusted their bets less to the chances of winning (see Fig. 4*a*). A similar relationship was observed in the IPC subregion, albeit at a level that did not reach significance (rho = −0.47, *P* = 0.11). The volume of damage in the SPC was not correlated with risk adjustment (rho = −0.13, *P* = 0.66). Overall betting and final point score were not significantly correlated with the extent of lesion in the pPAR ROIs (*P* > 0.1).

In the vmPFC group, overall betting was positively correlated with the volume of damage in the mOFC subregion (rho = 0.58, *P* = 0.04, *n* = 13; see Fig. 4*b*). Overall betting and the extent of damage in the mPFC were not correlated (rho = 0.04, *P* = 0.89), and the positive correlation between lesion volume in the overall vmPFC ROI and overall betting failed to reach statistical significance (rho = 0.44, *P* = 0.13). Risk adjustment and final point score were not significantly correlated with the extent of lesion in the vmPFC ROIs (*P* > 0.2).

### Response Times

Response times varied as a function of the choice condition (main effect: *F*_1,45_ = 16.65, *P* < 0.001, ηp^2^ = 0.27) and the chances of winning (main effect: *F*_2,90_ = 20.32, *P* < 0.001, ηp^2^ = 0.31), and a significant chances of winning by choice interaction were observed (*F*_2,90_ = 12.81, *P* < 0.001, ηp^2^ = 0.22, see Supplementary Fig. 3). There was a significant main effect of group (*F*_2,45_ = 3.20, *P* = 0.05, ηp^2^ = 0.12), with longer response times in the pPAR group than in healthy controls (*P* = 0.02, *d* = 0.85; pPAR vs. vmPFC: *P* = 0.29, *d* = 0.35; vmPFC vs. controls: *P* = 0.20, *d* = 0.67), but group status did not interact significantly with other task parameters (group × chances of winning: *F*_4,90_ = 0.60, *P* = 0.66; group × choice: *F*_2,45_ = 0.67, *P* = 0.52; 3-way interactions: *F*_4,90_ = 1.21, *P* = 0.31). Hence, the increased response times in the pPAR group are likely to reflect nonspecific slowing of motor responding and/or cognitive processing.

### Exploration of Learning Effects on the RBT

The design of the RBT presented full information about the available wins, losses, and probabilities on each trial, partly in order to alleviate any learning requirement. Nevertheless, we conducted 2 sets of control analyses to assess whether choice behavior on the RBT was shaped by learning effects. First, we tested whether betting behavior was influenced by the outcome on the previous trial (e.g. Thaler and Johnson 1990). Risk adjustment, overall betting, and decision latencies in active-choice trials were calculated trials after a win and after a loss separately, and analyzed using a 2 (prior outcome) × 3 (group) mixed-model ANOVA. No significant main effect of prior outcome was found for any measure (risk adjustment: *F*_1,45_ = 0.69, *P* = 0.41, ηp^2^ = 0.02; overall betting: *F*_1,45_ = 0.21, *P* = 0.65, ηp^2^ = 0.01; decision latencies: *F*_1,45_ = 0.85, *P* = 0.36, ηp^2^ = 0.02). Likewise, no significant interactions between prior outcome and group status were found (risk adjustment: *F*_2,45_ = 0.68, *P* = 0.51, ηp^2^ = 0.03; overall betting: *F*_2,45_ = 1.02, *P* = 0.37, ηp^2^ = 0.04; decision latencies: *F*_2,45_ = 0.98, *P* = 0.38, ηp^2^ = 0.04). A significant main effect of group for risk adjustment (*F*_2,45_ = 7.31, *P* = 0.002, ηp^2^ = 0.24) and overall betting (*F*_2,45_ = 3.59, *P* = 0.04, ηp^2^ = 0.14) corroborated the primary analyses reported above.

Secondly, we compared risk adjustment, overall betting, and decision latencies in active-choice trials in the first versus second half of the RBT, using 2 (task half) × 3 (group) mixed-model ANOVAs. No significant main effect of task half was found for risk adjustment (*F*_1,45_ = 0.16, *P* = 0.69, ηp^2^ = 0.01). Overall betting decreased marginally in the second half (*F*_1,45_ = 4.01, *P* = 0.05, ηp^2^ = 0.08), and decision latencies were shorter in the second half of the task (*F*_1,45_ = 30.54, *P* = 0.001, ηp^2^ = 0.40). No significant interaction between task half and group was found for any measure (risk adjustment: *F*_2,45_ = 0.06, *P* = 0.94, ηp^2^ = 0.01; overall betting: *F*_2,45_ = 0.70, *P* = 0.50, ηp^2^ = 0.03; decision latencies: *F*_2,45_ = 0.26, *P* = 0.77, ηp^2^ = 0.01). The main effect of group was significant for risk adjustment (*F*_2,45_ = 6.98, *P* = 0.002, ηp^2^ = 0.34) and overall betting (*F*_2,45_ = 3.87, *P* = 0.03, ηp^2^ = 0.15), but not for decision latencies (*F*_2,45_ = 2.25, *P* = 0.12, ηp^2^ = 0.09), again confirming the results obtained in the primary analyses.

In conclusion, consistent with the task's design as a test of decision-making under explicit risk, we saw minimal evidence of learning-related changes in choice behavior, and importantly, these effects did not differ between the groups.

### Performance on Control Tasks

No significant group effects were found on the dependent variables on the Beads Game (*P* > 0.1) or the Probability Adjustment Task (*P* > 0.4) (see Supplementary Table 1). Given that the Probability Adjustment Task was aborted in 3 pPAR lesion patients who were unable to adequately count the beads, the exclusion of these (evidently impaired) patients from the analysis may under-estimated the effects of pPAR damage on performance in this task. To examine whether the impairments on the RBT were fully dissociable from performance on the Probability Adjustment Task, a single-case assessment is presented in Supplementary Table 2 that corroborates the conclusion that, in both lesion groups, deficits in risk-sensitive decision-making can occur independently of the performance level on the Probability Adjustment Task. In a sensitivity analysis, the reduced risk adjustment on the RBT in the pPAR group remained significant when excluding the 3 pPAR patients with counting difficulties (see Supplementary Analysis 1).

## Discussion

In the current study, we assessed risk-sensitive choice behavior in patients with damage to the vmPFC and to the pPAR using the RBT. The present data provide the first evidence for the necessary role of both the areas in human decision-making under risk. The analysis of betting behavior on the RBT indicated the specific components of decision-making that are affected by pPAR and vmPFC damage. A reduction in risk adjustment was observed in both patient groups. This effect was correlated with the volume of damage in the pPAR region, but was not significantly related to the volume of vmPFC damage. Patients with damage to the vmPFC additionally chose higher bets overall compared with both the healthy control and the pPAR groups, and the overall betting measure was correlated positively with the volume of damage to the mOFC. Both lesion groups accumulated fewer points than healthy controls across the task. The pPAR group also manifested slower decision-making, as a general motor effect that did not interact with the task condition. In 2 control tasks that involved probabilistic reasoning outside of a gambling context, there was no evidence of significant impairment in either lesion group.

The finding that the pPAR lesion group displayed reduced risk adjustment was in line with our hypothesis: These patients failed to adequately adapt their bets to the likelihood of winning. Our data corroborate electrophysiological recordings in nonhuman primates, showing that pPAR activity during response selection reflects reward likelihood (Platt and Glimcher 1999; Shadlen and Newsome 2001; McCoy and Platt 2005; Yang and Shadlen 2007; Platt and Huettel 2008; Kable and Glimcher 2009; Kiani and Shadlen 2009). Previous neuroimaging data in humans also indicate that activity in the pPAR during decision-making under risk represents the probability and variance of outcomes (Huettel et al. 2005; Van Leijenhorst et al. 2006; Smith et al. 2009; Vickery and Jiang 2009; Symmonds et al. 2011; Studer et al. 2012). The present results provide an important extension of these previous findings with convergent techniques by confirming the causal contribution of pPAR in human decision-making. Our data further show that the degree of impairment in risk adjustment scales proportionately with the volume of pPAR damage.

Neuroeconomic models of decision-making often distinguish 2 stages of processing: A valuation stage, in which the subjective value of an option is established, and a choice stage, in which a response option is selected based on the input from valuation (e.g. Rangel et al. 2008; Kable and Glimcher 2009; Rangel and Hare 2010). In which of these stages might the pPAR be involved? The electrophysiological studies associate pPAR activity with the reward likelihood (and magnitude) attached a specific response, implementing the pPAR in the choice stage (Kable and Glimcher 2009). In a recent fMRI study of the RBT in healthy volunteers, we compared the sensitivity of pPAR responses with the odds of winning during active-choice versus no-choice trials, and found greater sensitivity under conditions of active choice, that is, when the probability information was used to guide bet selection (Studer et al. 2012). In the current study, we found that while patients with pPAR damage failed to use the information about the chances of winning adequately in their bet selection, the processing of probabilities outside of a gambling context (as assessed on the 2 control tasks) was largely unimpaired. Taken together, these results seem to implicate the human pPAR in the choice stage of the decision process, or as an interaction hub between valuation and choice circuitries. Future research should aim to test this directly.

The vmPFC lesion group displayed a distinct impairment, placing higher bets overall compared with both the healthy control and pPAR lesion groups. The necessary involvement of the vmPFC in successful decision-making is well established (e.g. Bechara et al. 1994, 1999, 2000, 2005; Fellows and Farah 2005; Weller et al. 2007; Floden et al. 2008), and the increase in risk appetite seen here replicates past studies using the Cambridge Gamble Task in groups with vmPFC pathology (Mavaddat et al. 2000; Manes et al. 2002; Clark et al. 2008). At the same time, the current data extend our understanding of this deficit in 2 important ways. First, a key modification in the design of the RBT is that the red/blue probability decision was removed, allowing the evaluation of betting behavior across trials with both positive and negative odds, that is, in conditions where risk-taking is advantageous and in conditions where risk-taking is disadvantageous (respectively). The elevation in betting in the vmPFC group was most apparent on the unfavorable (40%) condition, where conservative choice (i.e. low betting) is the optimal strategy. As such, vmPFC may ordinarily be most critical in situations, where conservative choice is most adaptive, rather than taking risks. Secondly, the increase in betting in the vmPFC lesion group was specifically associated with the extent of damage in the mOFC subregion. This is consistent with both neuroimaging data in humans and electrophysiological work in rodents and monkeys, implicating the mPFC region in the valuation of decision options (e.g. Wallis and Miller 2003; Padoa-Schioppa and Assad 2006; Plassmann et al. 2007, 2010; Chib et al. 2009; FitzGerald et al. 2009; Kang et al. 2011). Our correlational result also substantiates a similar reported relationship between the volume of mOFC damage and immediacy bias on delay discounting (Sellitto et al. 2010) and a study in patients with selective mOFC lesions, showing a specific effect on the maintenance of stimulus value following positive feedback (Camille et al. 2011).

Contrary to our predictions, a reduction in risk adjustment was also observed in the vmPFC group, comparable with the deficit in the pPAR lesion patients. This was an unexpected result, given that the previous studies with the Cambridge Gamble Task found comparable risk adjustment slopes in healthy controls and patients with vmPFC damage (Mavaddat et al. 2000; Manes et al. 2002; Clark et al. 2008). One pertinent difference in the RBT is the inclusion of a greater range of probabilities, including trials with negative odds, which may have unmasked a subtle deficit in the vmPFC group. But is it possible that impaired risk adjustment could be a nonspecific consequence of brain damage, akin to Lashley's mass action? This is unlikely in our opinion; first, our previous studies (Rogers et al. 1999; Manes et al. 2002; Clark et al. 2008) with the Cambridge Gamble Task have shown no abnormalities in either risk adjustment or overall betting in lesion control groups (with primarily dorsal frontal damage). Secondly, and more critically, while both lesion groups here showed reduced risk adjustment, the degree of impairment was only significantly associated with the volume of damage in the pPAR, but not in the vmPFC, or the overall lesion volume. Indeed, the similar performance between the vmPFC and pPAR groups was exclusively observed in the 40% condition, and when the odds reversed to being highly favorable (80% condition), lower betting was only observed in the pPAR group, who differed from both healthy controls (significantly) and the vmPFC patients (*P* = 0.07). Thus, while the present result does not constitute a full double dissociation between the vmPFC and pPAR lesion groups, a quantitative difference was clearly apparent in the risk adjustment profiles, and the ROI correlations further point to a greater degree of regional specificity than the basic group analysis.

Damage to the vmPFC and pPAR may also disrupt other cognitive functions, which might be relevant to performance on decision-making tasks. While the vmPFC is also implicated in reinforcement learning, and indeed other probes of decision-making may be directly confounded by learning impairments (e.g. Fellows and Farah 2005; Tsuchida et al. 2010), the RBT measures decision-making under explicit risk, that is to say outside of a learning context. Further, control analyses confirmed the minimal influence of previous feedback or time-on-task on betting behavior. Damage to the pPAR has been associated with biases in visuospatial attention (Driver and Mattingley 1998; Mesulam 1999; Vandenberghe and Gillebert 2009; Verdon et al. 2010), and while no signs of neglect or visual extinction were evident during testing in our sample (consisting of mainly left unilateral lesions), it is conceivable that some patients had subtle undetected biases in visuospatial attention. However, potential side biases are unlikely to contaminate choice behavior on our task, as the wheels were presented centrally with winning segments evenly distributed over both hemifields, and long, self-paced presentation periods were used. Finally, a deficit in risk adjustment could potentially arise from a disruption in lower-level numerical cognition, a domain that has been associated with the parietal cortex in particular (see Ansari 2008; Sandrini and Rusconi 2009; Arsalidou and Taylor 2011 for recent reviews). However, we observed no significant effects of vmPFC and pPAR lesions on 2 control tasks assessing probabilistic reasoning outside of a gambling context; that is to say, without the involvement of a bet and without gain/loss consequence. Interestingly, a recent imaging study of the Beads Game found that activity in the pPAR correlated with increased bead sampling across participants (Furl and Averbeck 2011). However, importantly and in contrast to our task, the version of the Beads Game used in this previous study entailed monetary rewards and losses and, indeed, the parietal response was also highly sensitive to the reinforcement contingency. Our results indicate that pPAR and vmPFC are primarily involved in probabilistic processing under risky conditions when gain/loss information must be incorporated into the choice.

The vmPFC and the somatosensory cortex, which was affected in some of our pPAR lesion groups, have been proposed as key regions in the activation of somatic markers (Damasio 1994; Bechara and Damasio 2005). Previous research indicates that these peripheral arousal signals can aid decision-making in reward-learning environments and are sensitive to prefrontal damage (Bechara et al. 1999, 2000, 2005). Based on this framework, one might speculate that the observed abnormalities in the choice behavior of vmPFC and pPAR lesion patients were not caused by the impairment of cognitive decision processes, but by the attenuation of psychophysiological responses forming a component of emotional decision processes. It would be interesting for future research to investigate whether peripheral responses act as decision input signals under explicit risk and record peripheral responses in lesion samples.

In conclusion, our data demonstrate that the pPAR and vmPFC are causally involved in risk-sensitive decision-making. Our results also provide new insights into the specific sensitivities of these regions in choice under risk. Increased overall risk appetite was apparent in vmPFC lesion patients, and this increased risk-taking was particularly prominent in conditions where choosing conservatively was the most adaptive strategy. The ability to adjust the bet to the probability of winning was disrupted by both pPAR and vmPFC lesions; however, the degree of this impairment was specifically associated with the volume of pPAR damage.

## Supplementary Material

Supplementary material can be found at: http://www.cercor.oxfordjournals.org/.

## Funding

This research was supported by a consortium award from the Medical Research Council UK (grant number G1000183) and the Wellcome Trust (grant number 093875/Z/10/Z) to the Behavioural and Clinical Neuroscience Institute. Funding to pay the Open Access publication charges for this article was provided by the Wellcome Trust and the Research Councils UK.

## Supplementary Material

Supplementary Data
