# Supplementary material for: Risk-Sensitive Decision-Making in Patients with Posterior Parietal and Ventromedial Prefrontal Cortex Injury
Source: Cereb Cortex. 2013 Aug 7;25(1):1–9. doi: 10.1093/cercor/bht197 (PMC4259274; doi:10.1093/cercor/bht197)
Supplement: Supplementary Data [file supp_25_1_1__index.html]

Risk-Sensitive Decision-Making in Patients with Posterior Parietal and Ventromedial Prefrontal Cortex Injury — Risk-Sensitive Decision-Making in Patients with Posterior Parietal and Ventromedial Prefrontal Cortex Injury — Supplementary Data 

# Risk-Sensitive Decision-Making in Patients with Posterior Parietal and Ventromedial Prefrontal Cortex Injury

## Supplementary Data

Supplementary Data

**Files in this Data Supplement:**

- Supplementary Data - Docx file
