# Supplementary material for: Risk-Sensitive Decision-Making in Patients with Posterior Parietal and Ventromedial Prefrontal Cortex Injury
Source: Cereb Cortex. 2013 Aug 7;25(1):1–9. doi: 10.1093/cercor/bht197 (PMC4259274; doi:10.1093/cercor/bht197)
Supplement: Supplementary Data [file supp_bht197_bht197supp.docx]

## Supplementary material

**Manuscript: *Risk-sensitive decision-making in patients with posterior parietal and ventromedial prefrontal cortex injury***

**Authors: Bettina Studer, Facundo Manes, Glyn Humphreys, Trevor W. Robbins, Luke Clark**


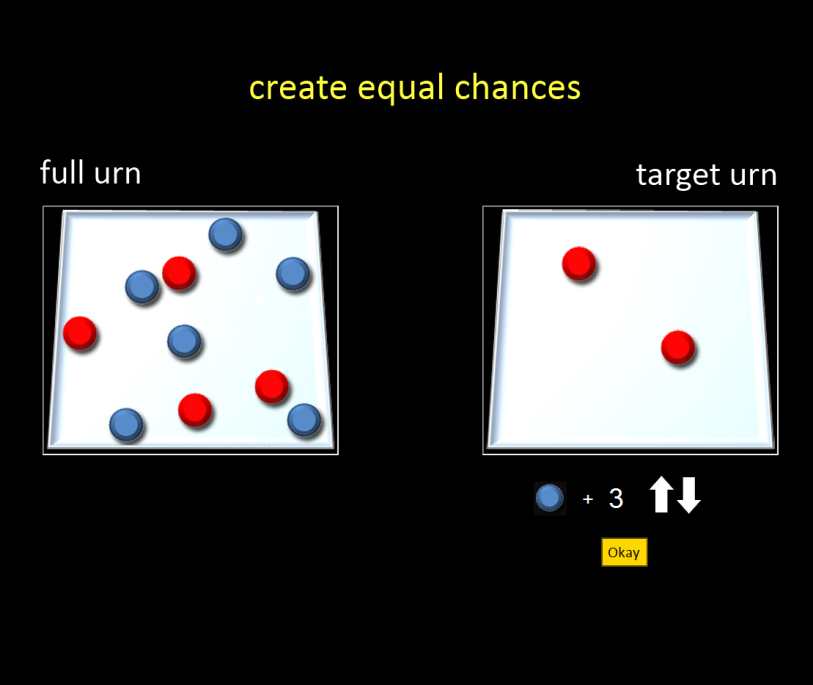


Supplementary Figure 1 – Probability Adjustment Task

On each trial, participants were asked to create equal chances of drawing a red bead in both urns, by adding blue beads to the target urn. In other words, participants were required to match the proportion of blue to red beads in the full urn.


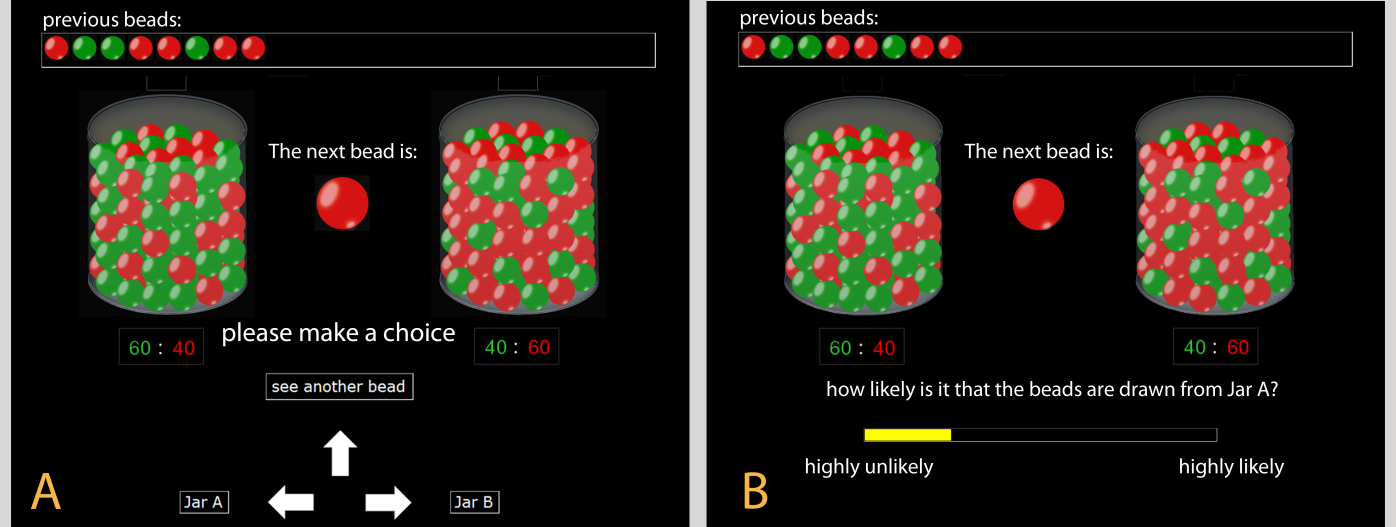


Supplementary Figure 2 – Beads Game

A: ‘Draws to decision’ - condition. Participants’ goal is to determine which jar the beads are taken from. After each draw, they can either ask for another bead or select Jar A or Jar B. B: ‘Probability estimates’ - condition. After each of 10 consecutive draws participants indicate the likelihood that the beads are being drawn from Jar A.


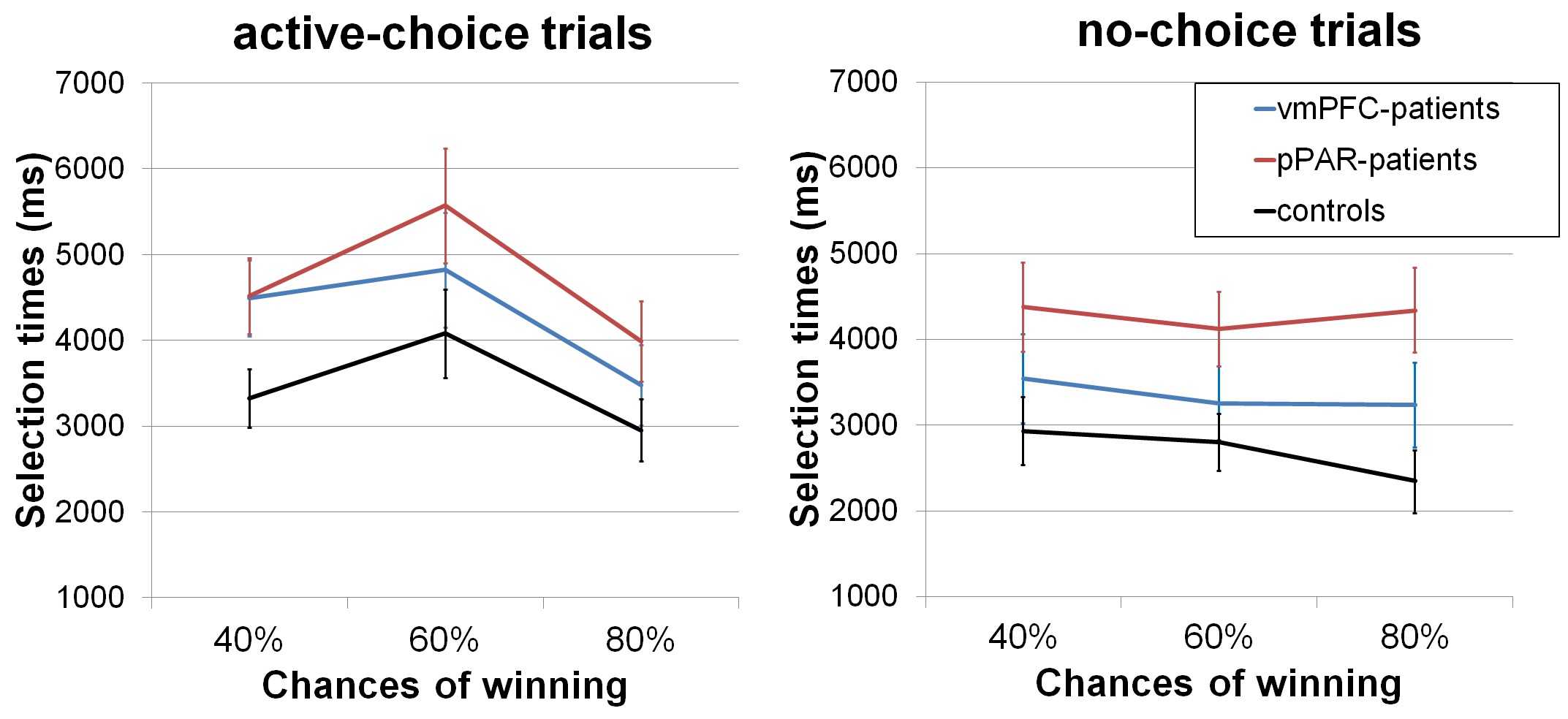


Supplementary Figure 3 - Decision latencies in active-choice and no-choice trials.

Selection times were higher in active-choice trials than no-choice trials (P<.001) and were modulated by the chances of winning (P<.001), particularly in the active choice conditions (interaction: P<.001). Individuals with lesions to the pPAR generally took longer to select a bet than healthy controls (P=.02), but this effect was independent of the chances of winning and the choice condition. Error bars represent SEM.

**Supplementary Table 1: Performance on control tasks.**

| **Probability Adjustment Task** | | | | | |
| --- | --- | --- | --- | --- | --- |
| **Performance** | **% correct answers**  **Mean (**±**SEM)** | | | **Main effect of group**  **(Kruskal-Wallis test)** | |
|  | **pPAR**  (n=8) | **controls**  (n=22) | **vmPFC** (n=11) | **Χ²** | **P** |
| overall | 73 (±26) | 68 (±32) | 61 (±31) | .64 | .72 |
| easy trials | 88 (±8) | 73 (±9) | 82 (±7) | .68 | .71 |
| medium trials | 88 (±6) | 73 (±7) | 70 (±11) | 1.22 | .54 |
| difficult trials | 50 (±18) | 61 (±9) | 39 (±13) | 1.67 | .43 |
| **Beads Game** | | | | | |
| **Measure** | **Mean (**±**SEM)** | | | **Main effect of group**  **(ANOVA)** | |
|  | **pPAR**  (n=9) | **controls**  (n=20) | **vmPFC**  (n=12) | **F** | **P** |
| *Ratio of beads in jars = 60 to 40* | | | | | |
| Number of drawn beads | 8 (±1.6) | 8 (±1) | 8 (±1.4) | .00 | .99 |
| Initial certainty | 55 (±7) | 62 (±3) | 60 (±3) | 1.23 | .30 |
| Final certainty | 55 (±3) | 55 (±2) | 52 (±4) | .28 | .78 |
| Response to disconfirmatory evidence | 25 (±6) | 18 (±3) | 28 (±5) | 2.06 | .14 |
| *Ratio of beads in jars = 85 to 15* | | | | | |
| Number of drawn beads | 6 (±1.5) | 5 (±1) | 5 (±1.3) | .10 | .91 |
| Initial certainty | 63 (±12) | 69 (±6) | 62 (±9) | .25 | .78 |
| Final certainty | 71 (±10) | 79 (±4) | 71 (±8) | .57 | .57 |
| Response to disconfirmatory evidence | 44 (±13) | 27 (±7) | 44 (±10) | 1.16 | .33 |

**Supplementary Table 2: Single Case Evaluation of performance on RBT and Probability Adjustment Task**

Performance of each lesion patients was compared against the average performance of healthy controls. Patients were classified as impaired on a measure if their score was at least one standard deviation below the mean of the healthy controls, and are marked in red. The displayed results show that several patients were impaired on one task, but unimpaired on the other (for both lesion groups). Thus, the analyses demonstrate that impairment on the RBT can be dissociated from impaired performance on the Probability Adjustment Task in both the pPAR lesion group and the vmPFC lesion group.

|  | **Roulette Betting Task** | **Probability Adjustment Task** | |
| --- | --- | --- | --- |
|  | *Final score* | *Overall performance* | *Performance difficult trials* |
| ***pPAR lesion patients*** | | | |
| P1 |  |  |  |
| P2 |  |  |  |
| P3 |  |  |  |
| P4 |  |  |  |
| P5 |  | *Aborted* | *Aborted* |
| P6 |  |  |  |
| P7 |  |  |  |
| P8 |  | *Aborted* | *Aborted* |
| P9 |  | *Not tested* | *Not tested* |
| P10 |  | *Not tested* | *Not tested* |
| P11 |  | *Aborted* | *Aborted* |
| P12 |  |  |  |
| P13 |  |  |  |
| ***vmPFC lesion patients*** | | | |
| F1 |  |  |  |
| F2 |  | *Not tested* | *Not tested* |
| F3 |  | *Not tested* | *Not tested* |
| F4 |  |  |  |
| F5 |  |  |  |
| F6 |  |  |  |
| F7 |  |  |  |
| F8 |  |  |  |
| F9 |  |  |  |
| F10 |  |  |  |
| F11 |  |  |  |
| F12 |  |  |  |
| F13 |  |  |  |

Supplementary Analysis 1

Three patients with pPAR lesions showed severe counting difficulties on the Probability Adjustment Control Task (P5, P8, P11). We examined whether impaired risk adjustment on the RBT was still apparent in the pPAR group if these three patients were excluded from the analysis (pPAR n=10, vmPFC n=13, healthy controls n=22). The results obtained in this analysis were identical to the results in the overall sample: a one-way ANOVA on risk adjustment confirmed a significant effect of group (F_2,44_=5.34, P<.01), with reduced risk adjustment in both lesion groups compared to healthy controls (pPAR vs. controls: P<.03, *d*=.90; vmPFC vs. controls P<.01, *d*=1.01; pPAR vs. vmPFC: P=.69, *d*=.14). Similarly, a significant group x chances of winning interaction was found in the ANOVA on bet size with chances of winning as a repeated-measures factor (F_4,84_=4.06, P<.01, ηp2=.16). The main effects of chances of winning (F_2,84_=138.84, P<.001, ηp2=.77) and group (F_2,42_=3.43, P<.05, ηp2=.14) were also confirmed. In conclusion, supplementary analysis 1 demonstrated that the impaired risk adjustment in the pPAR lesion group was not attributable to the inclusion of three patients with counting inability.
